# Loss of WNT2B Results in Epithelial Defects and Predisposes to Gastrointestinal Dysplasia in Humans

**DOI:** 10.1016/j.jcmgh.2025.101514

**Published:** 2025-04-11

**Authors:** Leslie Lori, Valentin Neuranter, Corinne Lebreton, Jérémy Berthelet, Marianna Parlato, Christina Michail, Anis Khiat, Dominique Berrebi, Julie Bruneau, Benoit Terris, Georgia Malamut, Sylvain Hanein, Yohann Schmitt, Céline Banal, Fernando Rodrigues Lima, Emilie Azouguene, Frank Ruemmele, Cecile Talbotec, Cecile Lambe, Nadine Cerf-Bensussan, Fabienne Charbit-Henrion

**Affiliations:** Université Paris-Cité, Institut Imagine, Laboratory of Intestinal Immunity, INSERM U1163, Paris, France; Université Paris Cité, CNRS, Epigenetics and Cell Fate, Paris, France; Université Paris-Cité, Institut Imagine, Laboratory of Intestinal Immunity, INSERM U1163, Paris, France; Université Paris Cité, CNRS, Epigenetics and Cell Fate, Paris, France; Université Paris-Cité, Institut Imagine, Laboratory of Intestinal Immunity, INSERM U1163, Paris, France; Department of Pathology, AP-HP. Centre-Université Paris Cité, Hôpital Necker-Enfants Malades, Paris, France; Department of Pathology, AP-HP. Centre-Université Paris Cité, Hôpital Cochin, Paris, France; Université Paris-Cité, Institut Imagine, Laboratory of Intestinal Immunity, INSERM U1163, Paris, France; Department of Gastroenterology, AP-HP. Centre-Université Paris Cité, Hôpital Cochin, Paris, France; Bioinformatic Platform, Institute of Genetic Diseases, INSERM UMR1163, Imagine, Université Paris-Cité and Structure Fédérative de Recherche Necker, Paris, France; Genomics Core Facility, Institut Imagine-Structure Fédérative de Recherche Necker, INSERM U1163 et INSERM US24/CNRS UAR3633, Paris Cite University, Paris, France; Université Paris-Cité, iPSC Core Facility, Institut Imagine, INSERM U1163, Paris, France; Université Paris Cité, CNRS, Unité de Biologie Fonctionnelle et Adaptative, Paris, France; Department of Genomic Medecine of Rare Diseases, AP-HP. Centre-Université Paris Cité, Hôpital Necker-Enfants Malades, Paris, France; Université Paris-Cité, Institut Imagine, Laboratory of Intestinal Immunity, INSERM U1163, Paris, France; Service de Gastro-entérologie et Nutrition Pédiatrique, AP-HP. Centre-Université Paris Cité, Hôpital Necker-Enfants Malades, Paris, France; Service de Gastro-entérologie et Nutrition Pédiatrique, AP-HP. Centre-Université Paris Cité, Hôpital Necker-Enfants Malades, Paris, France; Université Paris-Cité, Institut Imagine, Laboratory of Intestinal Immunity, INSERM U1163, Paris, France; Service de Gastro-entérologie et Nutrition Pédiatrique, AP-HP. Centre-Université Paris Cité, Hôpital Necker-Enfants Malades, Paris, France; Université Paris-Cité, Institut Imagine, Laboratory of Intestinal Immunity, INSERM U1163, Paris, France; Université Paris-Cité, Institut Imagine, Laboratory of Intestinal Immunity, INSERM U1163, Paris, France; Department of Genomic Medecine of Rare Diseases, AP-HP. Centre-Université Paris Cité, Hôpital Necker-Enfants Malades, Paris, France

WNT2B deficiency has been recently identified as a cause of congenital diarrhea, variably associated with ophthalmologic and/or endocrine features.^1^^,^^2^ WNT2B, produced primarily by subepithelial mesenchymal cells,^3^ signals through the β-catenin pathway, which is crucial for intestinal homeostasis and stem cell maintenance. In mice, β-catenin inactivation impairs intestinal stem cells maintenance and lead to fatal loss of intestinal function.^4^ Conversely, overactivation of WNT signaling occurs in 90% of colorectal cancers, causing abnormal epithelial proliferation.^4^ The redundancy of WNT2B with other Wnt ligands in mice highlights the need to study WNT2B-deficient patients to understand the disease mechanism.^5^ We report 2 female patients, P1 and P2, with severe neonatal diarrhea and failure to thrive, requiring parenteral nutrition (PN). Detailed clinical findings are summarized in the Supplementary Appendix. Of note, both patients developed severe osteoporosis and fractures from mild falls, a previously unreported feature. P1 and P2 carried compound heterozygous variants [NM_024494.2:c.409C>T:p.Arg137∗], and [c.794T>C:p.(Leu265Pro)] and a homozygous variant [NM_024494.2:c.681G>As:p.Thr227=] in *WNT2B*, respectively. The c.794T>C:p.(Leu265Pro) affected a highly conserved amino acid and was predicted to be damaging (Figure 1*A–B*; Supplementary Figure 1*A–B*). Prediction of splice donor loss for the c.681G>A:p.Thr227= synonymous variant was confirmed by cDNA sequencing (Figure 1*C*). Myc-tagged Arg137∗-WNT2B showed no expression in HEK293T cells (Supplementary Figure 1*C*), whereas the expression of the other 2 variants was not affected (Figure 1*D*). Their pathogenicity was therefore resolved using Alphafold2 to model the human WNT2B structure. WNT2B consists of an N-terminal domain containing a bundle of 12 α-helices (residues ∼1-270) and a C-terminal domain containing a two-stranded β-sheet (residues ∼340-380), connected by a large loop (Figure 1*E*, *right panel*). DYNAMUT predicted that the p.Leu265Pro variant destabilizes the central α-helix bundle (Figure 1*E*) by affecting the center of the α-helix H12 (residues 258-270). The 6-aminoacids insertion at Thr227 likely alters the interaction network between the 2 α-helices, H10 and H12, affecting the overall structure/function of the protein (Figure 1*F*). Confirming loss of WNT2B signalling, mRNA levels of WNT2B gene targets, *OLFM4*, *LGR5*, and *AXIN2*, were almost undetectable in gastric biopsies (Figure 1*G*). P1 and P2 presented severe gastric atrophy, partial duodenal villous atrophy, and glandular loss in the colon with moderate inflammatory infiltrate (Figure 1*H*; Supplementary Figure 2). Accordingly, plasma citrulline levels, a marker of enterocyte functional mass, were severely decreased in both patients (Supplementary Figure 3*A*).

**Figure 1:**
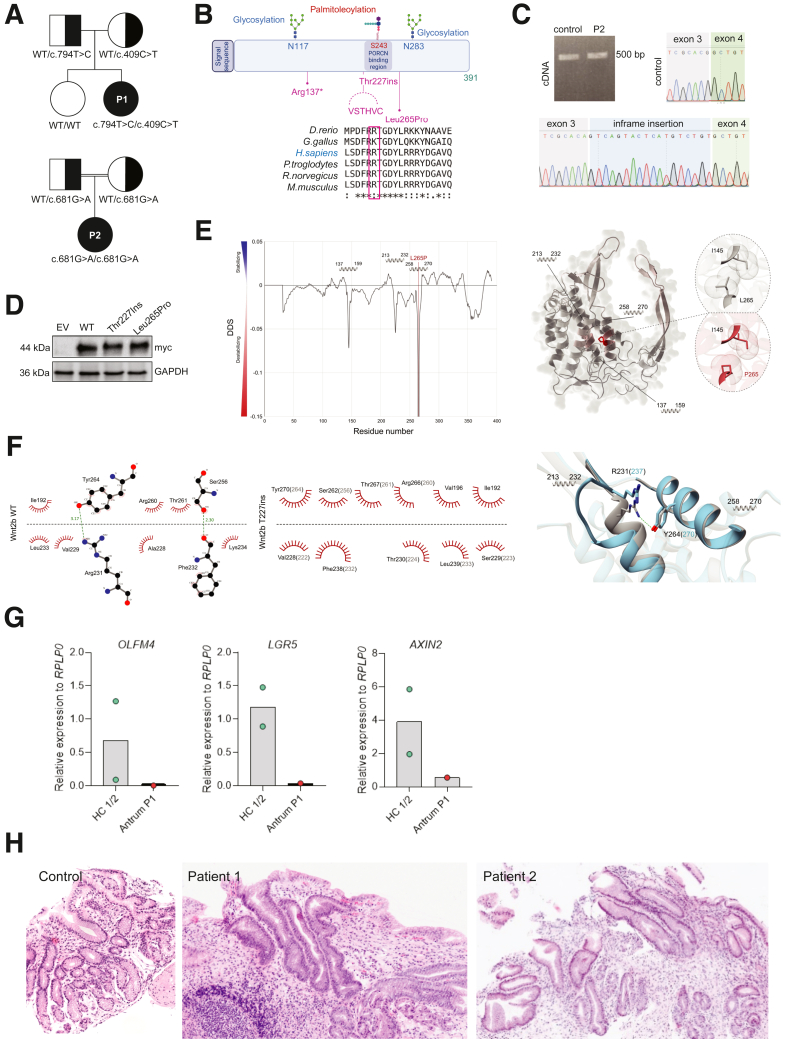
**WNT2B variants in P1 and P2 are loss of function and result in stem cell defect.** (*A*) Familial segregation of WNT2B variants. (*B*) Schematic representation of WNT2B protein; Inter-species conservation of Leu265 among orthologs. (*C*) Sequencing of cDNA synthetized from mRNA extracted from P2’s duodenal biopsy showing an 18-bp intronic sequence retention, resulting in a 6-amino acid insertion (VSTHVC). (*D*) Expression of Leu265Pro and Thr227ins variants in HEK293T cells transfected with pLenti-C encoding myc-WT-WNT2B, myc-Leu265Pro-WNT2B and myc-Thr227+6-WNT2B revealed by anti-myc antibody. GAPDH = loading control. pPURO-FLAG-HA-EGFP plasmid = transfection control. (*E*) *Left*: Stabilizing and destabilizing effects of the Leu265Pro-WNT2B variant, as well as the position of the α-helix bundles (137-159, 213-232, 259-270) are displayed. *Right*: Global structure of the Leu265Pro-WNT2B mutant. Regions with positive differences in vibrational entropy compared with WNT2B-WT are in *red* (level of red increases with the intensity of the difference). *Inset*: Close-up view of an interaction discrepancy caused by the Leu265Pro variant. WT and Leu265Pro are in *gray* and *red*, respectively. Van der Waals radii are shown for each side chain atom. (*F*) *Left*: Dimplots of the interactions between the α-helix bundle residues in the WT (*far left*) and Thr227ins mutant (*center*) (numbering of the WT-WNT2B residues in *gray*). Hydrophobic interactions are shown as *red cilia* and hydrogen bonds as *green dashed lines*. *Right*: Structural alignment of WT (*gray*) and Thr227ins (*blue*) WNT2B proteins. 213-232 and 258-270 α-helices, where the disappearance of the hydrogen bond between residues R231 and Y264, are shown in *green*. Numbering of the Thr227ins WNT2B mutant residues is displayed in *blue*. (*G*) *OLFM4*, *LGR5* and *AXIN2* mRNA expression by qRT-PCR in P1’s antral biopsy. (*H*) Patients’ antral biopsies, H&E staining, magnification 100×.

Impaired stem cell proliferation and atrophy do not fully explain the malabsorption in WNT2B-deficient patients. P1 showed significant nitrogen malabsorption (11% vs 54% energy absorption). Peptide absorption occurs via Na+-H+-linked amino-acid transporters, by maintening essential electrochemical gradients, in particular via the Na+/H+ exchanger NHE3 as its selective inhibition drastically reduces peptide uptake.^6^ Duodenal apical expression of NHE3 and DRA, a chloride/bicarbonate exchanger, was almost undetectable in both patients, unlike their normal expression in the colon. Other brush border enzymes including DPPIV, sucrose-isomaltase, and phosphoezrin were unaffected (Figure 2*A*; Supplementary Figure 3*B*). Further indicating epithelial abnormalities, paneth cells were mislocalized along the villous axis in both patients (Supplementary Figure 3*C*), as reported in Wnt ligands inducible knock-out mice.^5^ P1 showed prominent antral metaplasia, confirmed by MUC5AC overexpression (Figure 2*B, C, D*). In mice, WNT signaling drives early embryonic development of the gut, whereas its inhibition allows the development of stomach-specific epithelium.^7^ WNT ligands, through the transcription factor Cdx2, whose expression is completely restricted to the intestinal epithelium, activate large intestinal gene expression at high doses and small intestinal gene expression at lower doses.^7^ Thus, antral metaplasia might result from impaired WNT signaling in the small intestine causing default gastric-specific differentiation. Finally, intestinal organoids from patients’ induced pluripotent stem cells had defective 3D structures, with fewer having a single lumen compared with controls (Supplementary Figure 3*D, E*).

**Figure 2:**
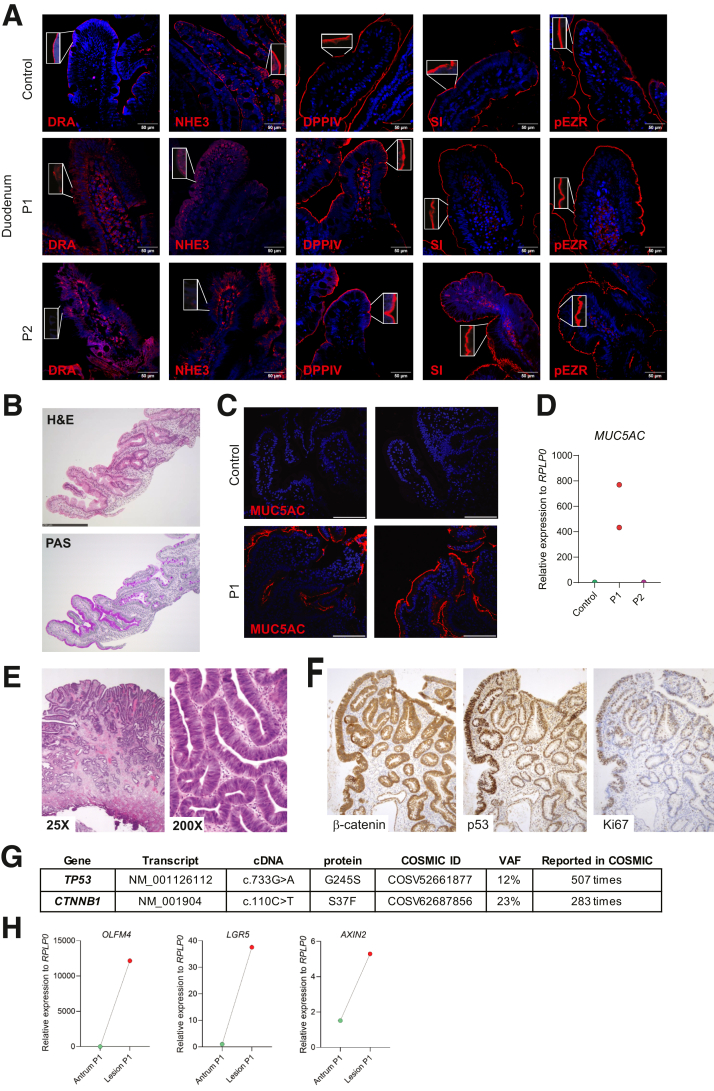
**WNT2B loss gives rise to an abnormal intestinal epithelial phenotype and predisposes to the emergence of somatic variants.** (*A*) DRA, NHE3, DPPIV, SI, and phospho-Ezrin expression in patients‘ duodenal biopsies by immunofluorescence (P1, age 9; P2, age 4 months old). DAPI: nuclei staining (*blue*); Scale bar = 10 μm. (*B*) Abnormal antral metaplasia in P1’s duodenal biopsy in H&E and PAS staining. (*C*) P1’s duodenal biopsy showing strong abnormal expression of gatric mucin MUC5AC. DAPI: nuclei staining, *blue*; Scale bar: 10 μm; confocal microcoscopy. (*D*) : Abnormal mRNA expression of *MUC5AC* in 2 distincts duodenal biopsies in P1, compared with P2 and healthy control. (*E*) H&E staining of antral adenoma with intestinal metaplasia and high-grade dysplasia with high-grade dysplasia, resected at age 10, low and high magnification. (*F*) Immunohistochemical staining of β-catenin, Ki67, and TP53 in same antral adenoma. (*G*) Main somatic variants identified by paired somatic-germline exome analysis. (*H*) *OLFM4*, *LGR5*, and *AXIN2* mRNA expression by RT-qPCR in non-dysplastic antrum of P1 compared with her precancerous lesion.

At age 9, an endoscopy in P1 incidentally revealed a pre-pyloric adenoma with metaplasia and low grade dysplasia, progressing to high-grade dysplasia (Figure 2*E, F*) and necessitating mucosal resection at age 13. High-grade dysplasia in a young patient with a stem cell proliferation defect was counterintuitive. Paired somatic-germline analysis revealed 2 main known-oncogenic variants, one in *TP53* hotspot conferring resistance to apoptosis^8^ and one in *CTNNB1*, encoding for β-catenin, a protein activated in presence of WNT ligand (Figure 2*G*). *CTNNB1* somatic variants are frequent in several human cancers.^9^ P1’s variant affected a key phosphorylation site Ser37,^9^ which is essential to trigger β-catenin degradation when WNT signaling is off. In P1, Ser37Phe, by stabilizing β-catenin, could reactivate the WNT/β-catenin signaling pathway in the adenoma. Accordingly, higher mRNA levels of *OLFM4*, *LGR5*, and *AXIN2* were found in the precancerous lesion compared with the non-dysplastic antrum (Figure 2*H*). The CTNNB1 variant acts as a somatic reversion of the monogenic defect, giving CTNNB1-mutant cells a proliferative advantage, similar to mechanisms in some hematopoietic cancers.^10^ Gastrointestinal malignancies have not been previously reported in WNT2B-deficient patients. However, these patients were much younger.^1^^,^^2^ Atrophy and stem cell defect may license selection of oncogenic somatic variants, stressing the need to monitor WNT2B-deficient patients for malignant transformation. WNT2B is not only critical for maintaining proliferation but also for proper development of intestinal epithelial cells. Our report could help anticipate the adverse effects of the WNT antagonists in development for the treatment of cancers.

## Supplementary Material

Supplementary Graphical Abstract
