# Supplementary material for: Loss of WNT2B Results in Epithelial Defects and Predisposes to Gastrointestinal Dysplasia in Humans
Source: Cell Mol Gastroenterol Hepatol. 2025 Apr 11;19(8):101514. doi: 10.1016/j.jcmgh.2025.101514 (PMC12288505; doi:10.1016/j.jcmgh.2025.101514)
Supplement: Supplementary Graphical Abstract [file mmc1.pptx]

## Slide 1
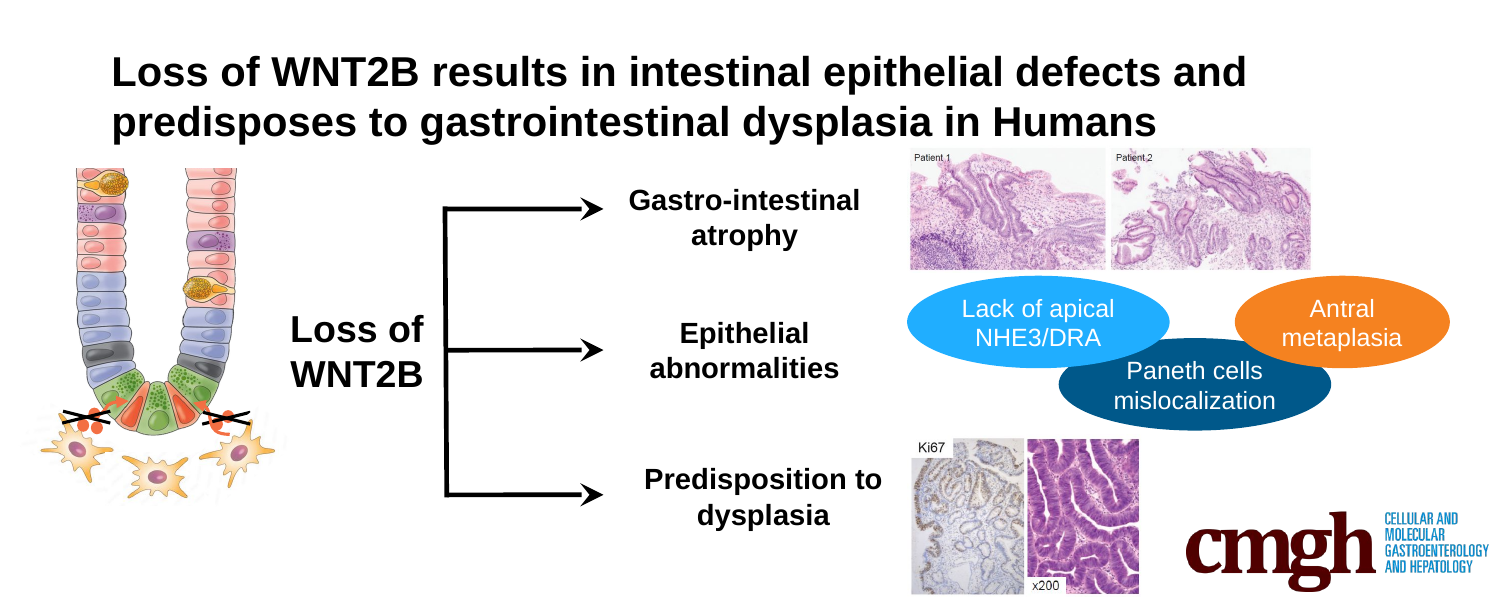

Loss of WNT2B results in intestinal epithelial defects and predisposes to gastrointestinal dysplasia in Humans
Gastro-intestinal atrophy
Lack of apical NHE3/DRA
Antral metaplasia
Loss of WNT2B
Epithelial abnormalities
Paneth cells mislocalization
Predisposition to dysplasia
