# Supplementary figures and images for: Loss of WNT2B Results in Epithelial Defects and Predisposes to Gastrointestinal Dysplasia in Humans
Source: Cell Mol Gastroenterol Hepatol. 2025 Apr 11;19(8):101514. doi: 10.1016/j.jcmgh.2025.101514 (PMC12288505; doi:10.1016/j.jcmgh.2025.101514)

# A

## WNT2B:c.409C>T

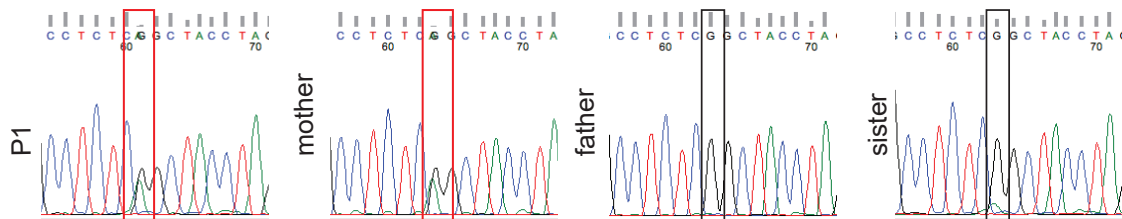

## WNT2B:c.794T>C

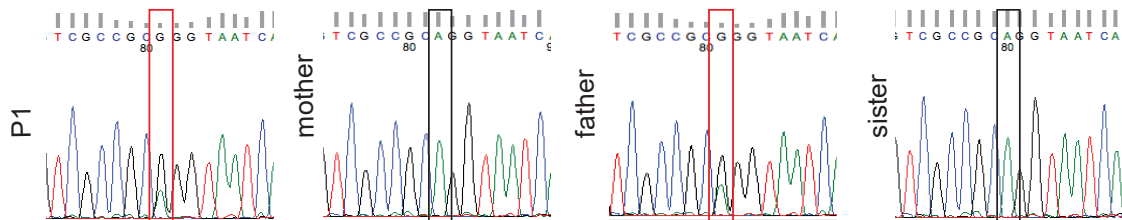

## WNT2B:c.681G>A

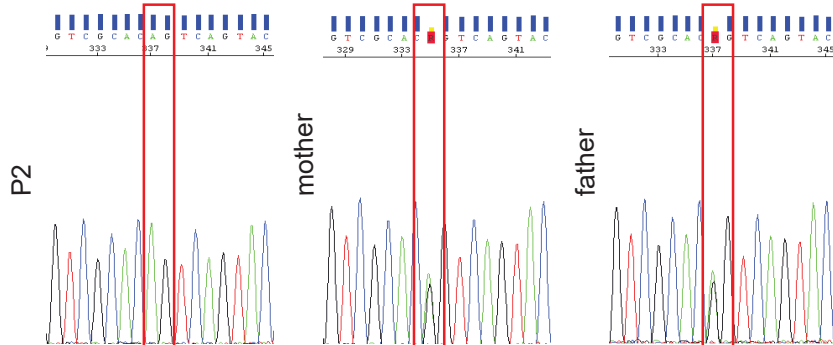

# B

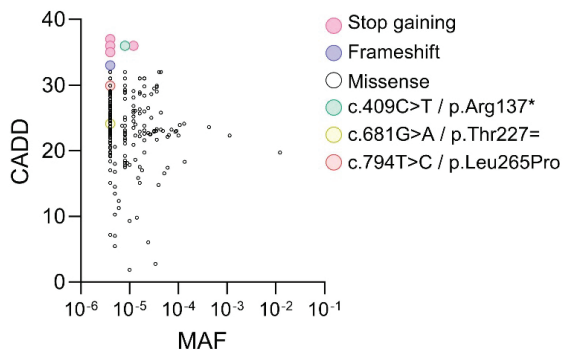

# C

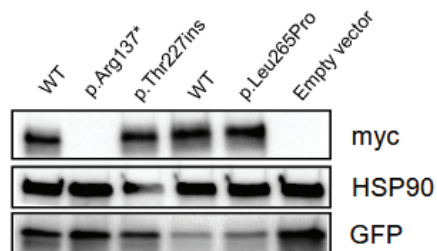

Supplement: Supplementary Figure 1 [file mmc2.pdf]

Patient 1

Colon

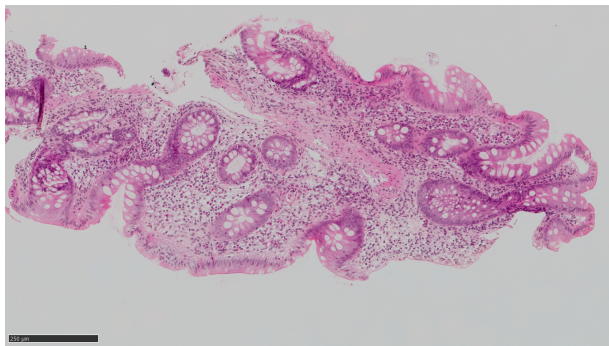

Patient 2

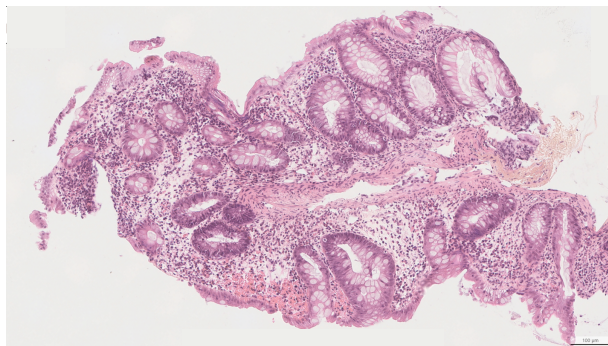

Duodenum

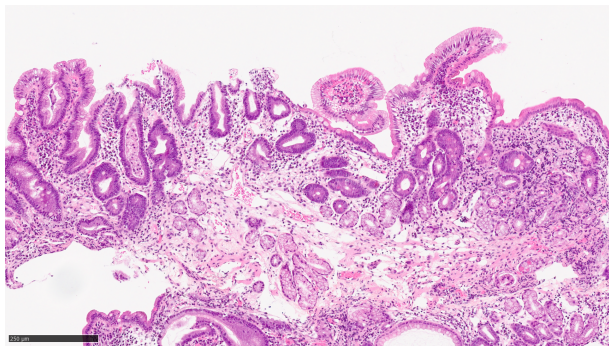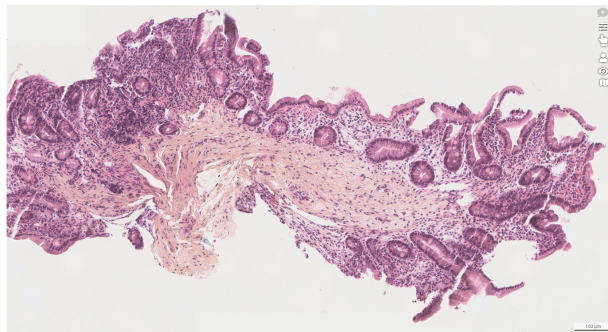

Stomach

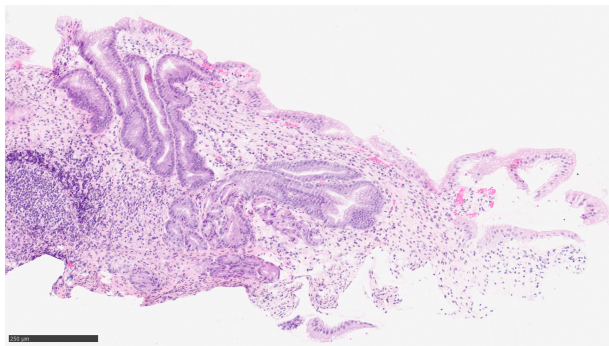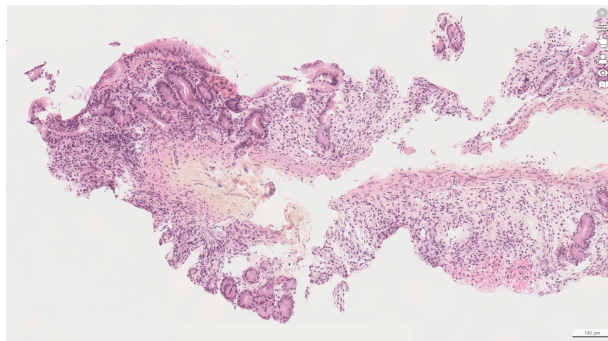

Supplement: Supplementary Figure 2 [file mmc3.pdf]

A Plasma citrulline levels (normal range 13-41  $\mu\text{mol/L}$ )

| P1 | 16,5 y | 15 y  | 14,5 y | 14 y | 13 y | 12 y | 11 y | 10 y |
|----|--------|-------|--------|------|------|------|------|------|
|    | 5      | 4     | 4      | 9    | 4    | 7    | 6    | 4    |
| P2 | 3 y    | 2,5 y | 2 y    |      |      |      |      |      |
|    | 3      | 2     | 2      |      |      |      |      |      |

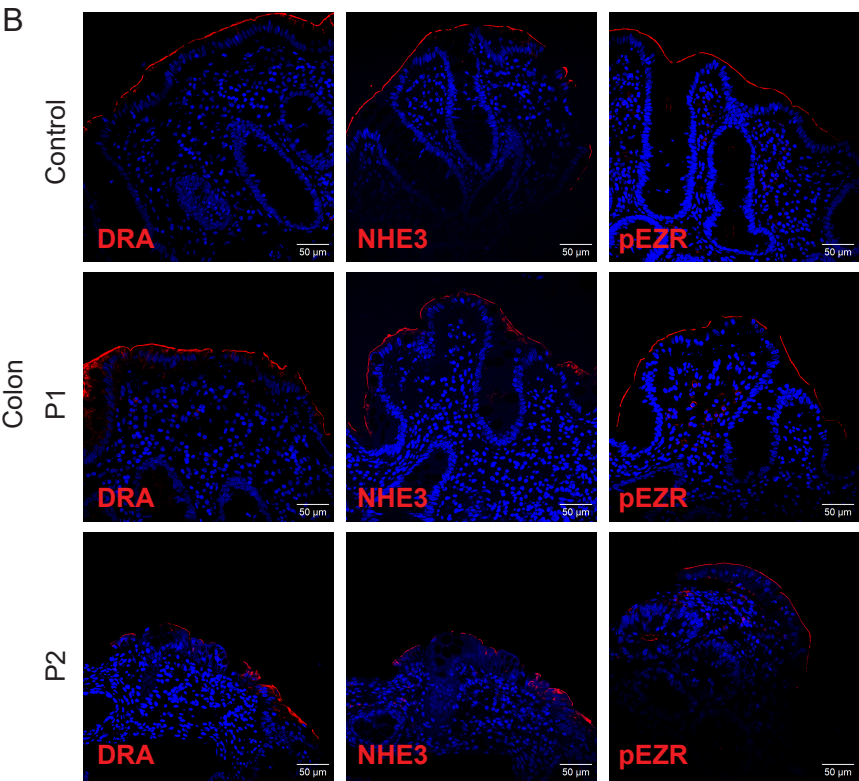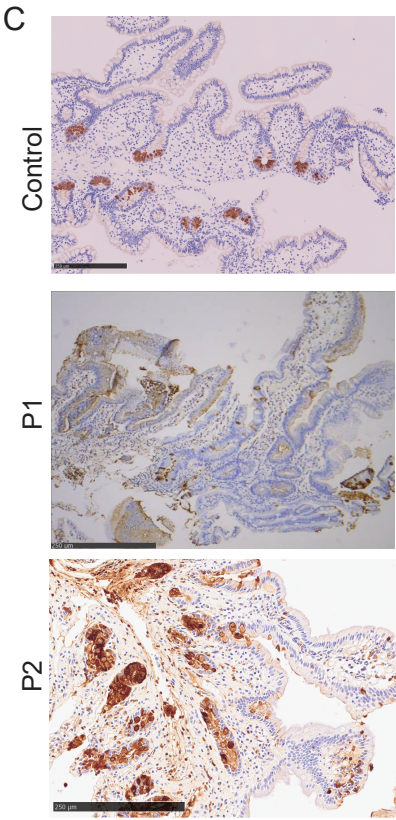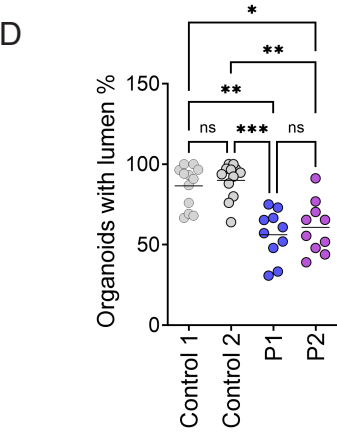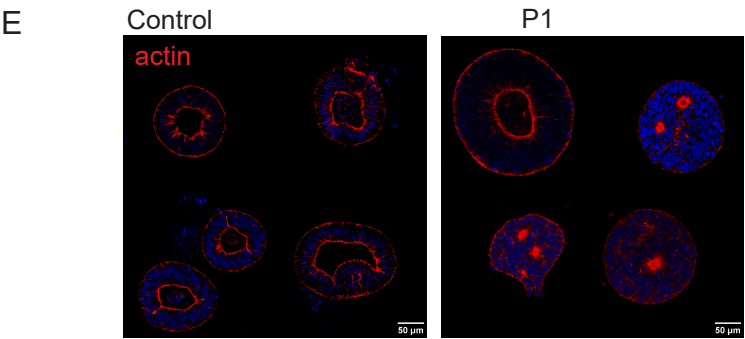

Supplement: Supplementary Appendix [file mmc4.pdf]
