# Supplementary material for: Loss of WNT2B Results in Epithelial Defects and Predisposes to Gastrointestinal Dysplasia in Humans
Source: Cell Mol Gastroenterol Hepatol. 2025 Apr 11;19(8):101514. doi: 10.1016/j.jcmgh.2025.101514 (PMC12288505; doi:10.1016/j.jcmgh.2025.101514)
Supplement: Supplementary Figure 3 [file mmc5.pdf]

Material and Methods

Patients' clinical data

P1, a girl born from non-related healthy parents in 2006, now aged 18, presented with severe diarrhea in the first days of life with 15% weight loss. Her older sister was healthy. Due to enteral feeding intolerance, severe diarrhea and failure to thrive, she was discharged on home parenteral nutrition (PN) at 6 months of age. PN was discontinued for 12 months at age 3 due to parental pressure, without dehydration or electrolytes disturbances, but growth stopped completely, leading to resume home PN. Over the years, her appetite and food tolerance were stable. Feces frequency and consistency did not change (about 5 liquid stools/day). Diagnosis of *WNT2B* deficiency was made in 2019. Her weight z-score fluctuated but overall improved from -2 to -1.5 without change in PN supply. At age 18, heir height was 155cm tall and weight 40 kg. She received home PN 5 nights a week, cycled over 12 hours, providing 1600ml and 1100 Kcal/day of non-protein energy over a week, which is 80% of caloric requirements for her age and weight. She was never admitted for dehydration or electrolyte disturbance. P1 does not have ophthalmological or gonadal abnormalities. She followed normal schooling until she was 14 year-old and then stopped to attend school because of hospital admissions and psychological issues. She was assessed as having a normal cognitive development.

P1 underwent repeated endoscopies in 2006 in 2008, and once or twice per year after 2015. Patient biopsies showed gastric atrophy and partial villous atrophy in the duodenum and in the colon with a moderate inflammatory infiltrate (Suppl Figure 2).

In 2015, at age 9, a stool absorption analysis was performed with the duplicate meal technique (11). Lipid malabsorption was the main abnormality with mild diarrhea, thus leading to investigate a lipid metabolism/absorption defect. To do so, dosage of lipase and co-lipase was performed in the duodenal secretions. During this upper gastro-intestinal tract endoscopy, a pre-pyloric mass was incidentally noted and biopsied. Histologic examination identified adenoma/intestinal metaplasia with low-grade dysplasia.

At age 12, the patient fell on her back at school and developed multiple vertebral compressions. Severe osteoporosis was confirmed by dual x-ray absorptiometry with z-score of -3.9. She benefited from bisphosphonate injections. Unfortunately, six months later, a third vertebral compression occurred after a mild shock on her back. In this context, she was admitted to a long-term rehabilitation center and started to use a wheel chair and a back brace. In total, she had three episodes of vertebral compression with low/no impact, due to severe osteoporosis. She is still treated by bisphosphonates infusions.

P2 is a younger girl born in 2021 from related parents (first cousins). She was born at at term (39 weeks), was small for gestational age (-2SD) with microcephaly. She displayed diarrhea with severe dehydration and failure to thrive in the first week of life and was started on PN. She was discharged on home PN at 6 months of age. At 3.5 years old, she was 87cm (-2SD) for 14kg (mean). She has about 10 liquid stools/day. She received home PN 7 nights a week, cycled over 14 hours, providing 80ml/kg/day and 1000 Kcal/day of non-protein energy over a week. P2 does not have gonadal abnormalities. As in patients previously described, P2's biopsies performed at 4 months of age showed atrophic gastritis and moderate inflammatory infiltrate in duodenum and colon (Suppl Fig 2). Unlike P1, P2 has ophthalmological abnormalities with bilateral microphtalmia associated with irian coloboma and microcornea in the left eye and sclerocornea in the right eye. She had multiple fractures since the age of 14 months with low/no impact (3 legs fractures, 1 arm fracture), and is treated by bisphosphonates infusions. She had no sign of tumorigenesis at latest endoscopy.

Study approval

P1 and P2 were recruited, with informed consent, at Necker Hospital as part of the Immunobiota Study ID-RCB NUMBER: 2014-A00017-40, with approval from Ethics Committee CPP-2014-01-04 and Competent Authority ANSM-140044B-42.

Next-generation and Sanger Sequencing

Next-generation sequencing were performed on genomic DNA extracted from P1's and P2's peripheral blood mononuclear cells (PBMC), using exome sequencing for P1 (Agilent SureSelect all exon V8) and targeted gene panel sequcing for P2 (Agilent Sureselect custom-made panel), as previously described (12). *WNT2B* variants were confirmed by Sanger sequencing using the primers:

Variant	Forward	Reverse
c.794T>C	TGGCGTGCACTCTCAGATTT	GACAAGATCAGTCCGGGTGG
c.409C>T	ACAGACATGGGCCTCTTTCC	GCACACACTCAGTTCACCCT
c.681G>A	GGGAAGAGGTTTCAGAGTCAG	GTTGGGGCTTTTGGAAGTG

RNA analysis

RNA was extracted from endoscopic biopsies obtained from both patients and pediatric controls using the RNeasy Plus Kit (QIAGEN, Courtaboeuf, France) according to the supplier's protocol. cDNA was obtained using M-MLV reverse transcriptase (Invitrogen) according to the

supplier’s protocol. Complementary DNA sequencing showing the VSTHVC insertion was performed using the following primers : Forward-GCCGTGTCATGCTCAGAAGT ; Reverse-CGGTGAAGTTGGCACCAT. Quantitative PCR was performed using Taqman™ Gene Expression Assay (Applied Biosystems™, no. 4331182) and Master Mix Taqman™ Universal II with UNG (Applied Biosystems™, no. 4440038). Results were normalized to *RPLPO* housekeeping gene.

Gene	Reference
AXIN2	Hs00610344_m1
LGR5	Hs00969422_m1
OLFM4	Hs00197437_m1
RPLPO	Hs00420895_gH

Cell Culture, Transfection, and Immunoblotting

The Lenti-X™ 293T Cell Line (Clontech) was cultured at 37°C in DMEM GLUTAMAX (Invitrogen) containing 10% FCS, and penicillin and streptomycin (100 U/ml each; Invitrogen). For transfection, Lenti-X™ 293T cells were plated in T-25 flask 24 h before transfection with with 4 µg of empty vector, WT-WNT2B and WNT2B-mutant alleles in presence of lipofectamine 2000 (Life Technologies). The pPURO-FLAG-HA-EGFP (6104bp) plasmid served as transfection control. 24 h post-transfection, cells were harvested after washing with ice-cold PBS and then lysed with ice-cold RIPA buffer supplemented with protease inhibitors (Roche). Cell lysates were separated by 4−15% SDS-PAGE gels (Bio-Rad Laboratories), then transferred to polyvinylidene difluoride membranes using the Trans-Blot Turbo Blotting System (Bio-Rad Laboratories). After blocking with 5% skim milk for 1 h, membranes were incubated with anti-HPS90 (#4874 Cell Signaling), anti-GAPDH (#14C10 Cell Signaling), anti-myc tag (#2278, Cell Signaling), anti-GFP (#2037, Cell Signaling) antibodies overnight at 4°C. Membranes were then incubated with anti-mouse (#7076, Cell Signaling) or anti-rabbit (#7074, Cell Signaling) antibodies conjugated with HRP for 1 h at room temperature and visualized using Clarity™ Western ECL Substrate (Bio-Rad) and the ChemiDoc XRS+ imaging system (Bio-Rad).

Site-directed Mutagenesis

The WT Human WNT2B (NM_024494) cDNA into pCMV-HA (#024494) (Origene, Rockville, USA), was used to generate Myc-tagged WNT2B variants by site-directed mutagenesis, using

GENEART® Site-Directed Mutagenesis System (Invitrogen, ThermoFisher Scientific) with the following primers:

Wnt2B_c.794T>C	Forward	gcacaggtgattacccgcggcgacactatga
	Reverse	tcatagcgtcgccgcgggtaatcacctgtgc
Wnt2B_ c.409C>T	Forward	atgctcagaagtagctgagaggcagcttttg
	Reverse	caaaagctgcctctcagctacttctgagcat
Wnt2B_c.682ins	Forward	acataataaccgctgtggtcgcacggtcagtactcatgtctgtgctgtgcggcggtttctgaagctgg
	Reverse	ccagcttcagaaaccgccgcacagcacagacatgagtactgaccgtgcgaccacagcggttattatgt

All WNT2B mutants were confirmed by Sanger sequencing.

Protein modeling

The AlphaFold model of human wild-type, p.L265P and p.T227_A228insVSTHVC WNT2B variants were computed by AlphaFold (AlphaFold Identifier AF-Q93097-F1; PDB code A4pN) (13). These models are of very high confidence with pLDDT scores for residues 70-385 higher than 90. The Dynamut protein stability server was used to analyze the p.L265P WNT2B variant (14). p.T227_A228insVSTHVC WNT2B variant was compared with the wild-type WNT2B model by dimplot using LigPlot software (15) and UCSF Chimera (16).

Human iPSC derived intestinal organoids

Intestinal organoids were generated from induced pluripotent stem cell (iPSC) lines derived from 2 healthy donors (one male age 38 years old and one female age 29 years old) (17) and P1 and P2. All lines were cultured in mTeSR 1 medium (Stem Cell Technologies) and transferred from a 60 mm petri dish to a 24-well plate coated with 3% hESC Matrigel (356231, Corning Inc.). To generate intestinal organoids, iPSC colonies were dissociated into clumps using Gentle Cell Dissociation Reagent (07174, Stem Cell Technologies) and replated in Matrigel-coated 24-well tissue culture plate (6×10^3 clumps per well) in mTeSR1. Cells were then differentiated into definitive endoderm over 3 days in Definitive Endoderm (DE) Medium (STEMdiff Endoderm Basal Medium [no. 05111] + STEMdiff Definitive Endoderm Supplement CJ [no. 05113], Stem Cell Technologies). The cells were then cultured in mid- hindgut (MH) medium (STEMdiff Endoderm Basal Medium [no. 05111] + STEMdiff Gastrointestinal Supplement PK [no. 05141] + STEMdiff Gastrointestinal Supplement UB (no.05142], Stem Cell Technologies) and free-floating mid- and hindgut spheroids appeared after 5 to 9 days of differentiation. Spheroids were then embedded in Matrigel domes and cultured in STEMdiff

Intestinal Organoid Growth Medium (OGM) (STEMdiff Intestinal Organoid Basal Medium [no. 05111] + STEMdiff Intestinal Organoid Supplement [no. 05144] + l-Glutamine, Stem Cell Technologies), allowing the differentiation of organoids in 20 days. Organoid cultures were passaged every 7 to 10 days based on density, size, and morphology.

Immunohistochemistry

Hematoxylin, and eosin (H&E), Periodic Acid Schiff (PAS), and specific immunohistochemistry stainings were performed in the laboratory of Pathology of Necker Hospital, according to manufacturers' protocols.

Target	Reference	Manufacturer
β-catenin	610154	BD Biosciences
Ki67	M7240	Dako
Lyzozyme	A0099	Dako
P53	M7001	Dako

Immunofluorescence and imaging

Paraffin sections (5 µm) were dewaxed in xylene and rehydrated in graded alcohol followed by 2 washes in PBS. Heat-induced antigen retrieval was carried out in Target Retrieval Solution citrate buffer pH 6(Dako, Agilent Technologies). Nonspecific binding was blocked by incubating in 3% BSA (Sigma-Aldrich) in PBS for 1 hour at room temperature (RT). Immunofluorescence of intestinal organoids was performed in IBIDI-8 wells plates. Organoids were fixed with 4% paraformaldehyde (PFA) for 1 hour at RT, permeabilized with PBS added with 0.2% triton X-100, and then blocked in 3% BSA for 1 hour. After staining the organoids are cleared with the RapiClear® 1.47 solution (Sunjin Lab). Stainings were performed with the following reagents, according to manufacturers' protocols:

	Targets	Reference
DAPI (NucBlue)	Nucleus	Invitrogen R37606
DRA	$Cl^-/HCO3^-$ exchanger	Abcam ab83545
NHE3	Na^+/H^- exchanger 3	Novus Biologicals NBP1-82574
DPPIV	Dipeptidyl peptidase 4	Novus Biological NBP1-84450

SI	Sucrase-isomaltase	Sigma WH0006476M1-100µg
Phospho-Ezrin	Phosphorylated Ezrin	Invitrogen PA5-37763
Phalloidin	Actin F	Invitrogen A12380
MUC5AC	Gastric mucin	Abcam ab3649

Imaging was performed on a confocal microscope (TCS SP8, Leica Microsystems, Nanterre, France), images were acquired with an 40 x 1.3 objective (HC PL APO, oil immersion, Leica Microsystems) and analyzed with Image J software from Fiji (https://imagej.nih.gov/ij/index.html).

Paired somatic-germline exome sequencing

Genomic DNA was extracted from peripheral mononuclear cells and from paraffin-embedded fragments obtained from the resected gastric adenoma with evidence of high grade-dysplasia in the Department of Pathology of Cochin Hospital. Exome libraries were prepared with the Twist Bioscience kits (Twist Human RefSeq Exome Kit, 36 Mb) and with the protocol version Twist-NGS Exome-96-12-DOC-001016-Rev1.0-May2018 on the Genomic Platform of Imagine Institute, according to the manufacturer protocols. Barcoded exome libraries were pooled and sequenced with the NovaSeq6000 system (Illumina), generating paired-end reads (100 bases + 100 bases). After demultiplexing, sequences were aligned to the reference human genome hg19 using the Burrows-Wheeler Aligner. The mean depth obtained was 285X with >97% of the targeted exonic bases covered by at least 30 independent reads. Downstream processing was carried out with the Genome Analysis Toolkit (GATK), SAMtools, and Picard, following documented best practices (http://www.broadinstitute.org/gatk/guide/topic?name=best-practices). To identify somatic mutations, analysis selected exonic or splice variants with low frequency in the gnomAD database (less than 5‰). Only variant with allelic fraction greater than 5%, and listed as pathogenic in Cosmic, Cancer hotspot, OncoKB or Cbioportal databases, were retained.

Graphs and statistical analyses

Data were analyzed using GraphPad Prism, version 10 (GraphPad Software, Inc, San Diego, CA). One-way ANOVA followed by Kruskal-Wallis multiple comparisons test was performed when appropriate. Changes were considered as statistically significant if P was less than 0.05.

All authors had access to the study data. They all reviewed and approved the final manuscript.

Supplementary References

11. Lacaille F, Vass N, Sauvat F, Canioni D, Colomb V, Talbotec C, et al. Long-term outcome, growth and digestive function in children 2 to 18 years after intestinal transplantation. Gut. avr 2008;57(4):455-61.

12. Charbit-Henrion F, Parlato M, Hanein S, Duclaux-Loras R, Nowak J, Begue B, et al. Diagnostic Yield of Next-Generation Sequencing in Very Early-Onset Inflammatory Bowel Diseases: A Multicenter Study. J Crohns Colitis. 18 mai 2018;

13. Jumper J, Evans R, Pritzel A, Green T, Figurnov M, Ronneberger O, et al. Highly accurate protein structure prediction with AlphaFold. Nature. août 2021;596(7873):583-9.

14. Rodrigues CH, Pires DE, Ascher DB. DynaMut: predicting the impact of mutations on protein conformation, flexibility and stability. Nucleic Acids Res. 2 juill 2018;46(W1):W350-5.

15. Laskowski RA, Swindells MB. LigPlot+: multiple ligand-protein interaction diagrams for drug discovery. J Chem Inf Model. 24 oct 2011;51(10):2778-86.

16. Pettersen EF, Goddard TD, Huang CC, Couch GS, Greenblatt DM, Meng EC, et al. UCSF Chimera--a visualization system for exploratory research and analysis. J Comput Chem. oct 2004;25(13):1605-12.

17. Quelennec E, Banal C, Hamlin M, Clémantine D, Michael M, Lefort N. Generation of two induced pluripotent stem cell lines IMAGINi004-A and IMAGINi005-A from healthy donors. Stem Cell Res. oct 2020;48:101959.

Supplementary Figures

Supplementary Figure 1 :

(A): Segregation analysis by Sanger sequencing. Electropherograms showing familial segregation of WNT2B mutations in P1's and P2's families.

(B): Frequency and CADD score for missense (white), stop-gain (pink) and predicted LOF (pLOF, purple) WNT2B variants reported in the gnomAD database and WNT2B variants reported in our 2 patients (c.409C>T green, c. 794T>C orange, C681G>A yellow). CADD : Combined Annotation Dependent Depletion ; MAF : Minor allele frequency

(C) : Absence of expression of Arg137* WNT2B variant, after transfection of HEK293T cells with complementary DNAs encoding Myc-tagged WT- WNT2B, Leu265Pro- WNT2B, Thr227+6-WNT2B, and Arg137*-WNT2B, and revelation by anti-myc antibody. Anti-HSP90 is used as loading control. A pPURO-FLAG-HA-EGFP plasmid served as transfection control.

Supplementary Figure 2 :

Standard biopsies in H&E staining, showing:

In P1:

- Colon : pseudo-villous appearance of the colon mucosae and slight crypt distorsion, with inflammatory infiltrate rich in eosinophils (age 9 years old)
- Duodenum : partial villous atrophy with extensive gastric metaplasia (age 16 years old)
- Stomach : hyperplastic and branching crypts with severe glandular atrophy and mononuclear inflammatory infiltrate (age 17 years old)

In P2 (all biopsies sampled at 4 months of age):

- Colon: surface epithelial damage with detachment, atrophy and crypt distortion , with focal mucin depletion and increase of apoptosis and inflammatory infiltrate in lamina propria rich in plasma cells and eosinophils with scattered neutrophils
- Duodenum: partial villous atrophy, slight crypt depletion and inflammatory infiltrate in lamina propria
- Stomach: severe glandular atrophy and slight inflammatory rich in eosinophils

Supplementary Figure 3:

(A) : Plasma citrulline levels for each patients, sampled at multiple time-points.

(B) : Normal expression of DRA, NHE3 and phosphoezrin in colonic biopsies from patients. DAPI: nuclei staining, blue; Scale bar: 10µm; confocal micrococoscopy. DRA : DownRegulated in Adenoma, encoded by *SLC26A3* ; NHE3 : Sodium/Hydrogen exchanger 3, encoded by *SLC9A3* ; pEZR : phosphoezrin.

(C) : Immunohistochemical staining of lysozyme showing abnormal localization of Paneth cells all along the villi in P1' and P2's duodenum.

(D-E) Single-lumen organoids counted in 4 independent organoid differentiations (passages 1 and 2). Nuclei=DAPI (blue); actin=phalloidin AF455 (red). Scale bar=10µm. Kruskal-Wallis multiple comparisons test.
